# Ensuring diagnostic testing accuracy for patient care and public health- COVID-19 testing scale-up from an EQA provider’s perspective

**DOI:** 10.1371/journal.pgph.0001615

**Published:** 2023-12-06

**Authors:** Veronica Restelli, Selvarani Vimalanathan, Mahfuza Sreya, Michael A. Noble, Lucy A. Perrone

**Affiliations:** Canadian Microbiology Proficiency Testing Program (CMPT), Department of Pathology and Laboratory Medicine, The University of British Columbia, Vancouver, British Columbia, Canada; Management and Science University, MALAYSIA

## Abstract

In response to the coronavirus pandemic (COVID-19) and scale up of diagnostic testing, the Canadian Microbiology Proficiency Testing program created a new proficiency testing (PT) program for the molecular and antigen detection of SARS-CoV-2. The program was geared to point of care testing (POCT) sites located in each of the eight provincial Health Authorities across British Columbia, Canada, with the intention to monitor testing quality. The PT program consisted of 6 shipments in a year, each containing a set of 4 samples either positive for SARS-CoV-2 virus or negative. The program began with initial 23 sites enrolling in March 2021, expanding to >100 participants by December 2021. After the first two surveys, it was observed that testing performance (accuracy) was consistently acceptable for sites using nucleic acid technology (NAT), however performance by sites using rapid antigen detection (RAD) methods was poor, especially when testing the weakly positive samples. A root cause investigation of poor testing performance revealed gaps in the execution of testing methods and also in results interpretation. These quality issues were most commonly associated with new testers who lacked experience with diagnostic testing. Tester training and mentoring was reinforced as was retraining of personnel; sample processing instructions were modified, and a training video was also created for testing sites. As a result of these interventions, sites improved their testing accuracy and the performance of POCT sites using RAD methods came to more closely match the performance of sites utilizing NAT. Overall, the PT program was highly successfully and improved quality of testing in the province. This work demonstrates the critical value of an external quality assessment (EQA) partner towards improving patient and public health and safety, especially when testing is conducted outside of an accredited medical laboratory setting.

## Introduction

In response to the 2019 severe acute respiratory syndrome coronavirus 2 (SARS- CoV-2) outbreak in China and subsequent global pandemic in 2020, the unprecedented rapid development, authorization, and implementation of novel in vitro diagnostics (IVDs) followed by large volume testing proliferated in nearly every nation in the world [1]. For some countries like Canada, for the first time in the history, diagnostic testing for both patient care and public health surveillance was approved for multiple commercial testing platforms and testing of patient samples was authorized to be performed outside the accredited clinical laboratory setting [2]. The resulting incidence and prevalence data informed (and in many cases directed) patient care and public health. This data also significantly influenced public health and local policy [3] at every level of society, and Canada was no exception.

Nucleic acid tests (NATs) were adopted as the laboratory gold standard for SARS-CoV-2 detection due to their high sensitivity and specificity, however the scale up of viral antigen testing became an essential tool for community-based testing and surveillance during the widespread pandemic. Between February 2020 and December 2022 there were 4,440,839 test confirmed cases in Canada and there have been 48,645 coronavirus disease (COVID-19) related deaths [4] as of December 21, 2022. In the province of British Columbia (BC) (population = 5,319,324 as of July 1, 2022) [5] there were 390,626 test confirmed cases and 4,715 related deaths. For the very first time, testing was being performed in long term care homes, primary schools, airports, and other facilities of patient care managed across the 8 different Health Authorities (HA). Most of these facilities were provided with government approved rapid antigen detection (RAD) assays or rapid nucleic acid detection (NAT) detection systems. These assays were classified as “low-complexity” defined as not requiring special technical knowledge to use, and as such could be performed by non-laboratory professionals. Studies have shown a sensitivity and specificity of 78.2% and 99.5% for RAD assays respectively; however higher sensitivities (up to 92.6%) have been reported in symptomatic individuals with positive viral culture [6–8]. The quick turnaround time of these assays were intended to enable rapid identification and isolation of infectious persons and prevent community spread.

In June 2020, British Columbia’s Provincial Health Services Authority mandated [9] that every facility conducting testing should be accredited. Each province in Canada is responsible for setting the regulatory process for diagnostic testing; in BC, the Diagnostic Accreditation Program (DAP) [10], is responsible for regulating diagnostic testing in the province. One of the requirements for testing site accreditation is the participation in an external quality assessment (EQA) program for every clinical diagnostic test performed, including COVID-19 testing.

EQA programs are an integral element of strong laboratory and health systems because they provide an objective service of assessment in accordance with international standards of quality [11]. For the last 10 years, in the case of medical laboratories, that international standard has been ISO 15189:2012 [12] (recently updated in 2022) and participation in EQA programs for each test in the laboratory service catalog is a requirement for accreditation to this standard. In order to satisfy the need for EQA of COVID-19 testing in BC and Canada, the Canadian Microbiology Proficiency Testing (CMPT) Program in collaboration with BC Centre for Disease Control (BCCDC) created a COVID-19 proficiency testing (PT) program oriented to community-based point of care testing (POCT) sites with support and enrollment also open to medical laboratories.

From January to March 2021, CMPT conducted research and development on appropriate challenge formulations and held consultation with health authorities and DAP to appropriately inform the design of the new EQA program. This program design was also informed by 40 years of CMPT experience as an ISO 17043 accredited EQA provider and by a panel of medical laboratory experts involved in patient care and public health across Canada. CMPT completed program development, validation, and testing site enrollment in early 2021 and shipped the first PT challenges in March 2021. Participation in the PT program quickly expanded from an initial 23 testing sites in March 2021 to >100 sites by March 2022 and eventually came to include many public and private laboratories and POCT sites as mandatory testing expanded in the province of BC and Canada. Available technologies and testing instruments also started to proliferate and CMPT adapted its PT samples to fit the needs of the participants and changing testing methods.

We present here nearly two years’ worth of COVID-19 EQA program implementation experience and information, and submit recommendations to the medical community reinforcing the unwavering value of ongoing EQA as an essential activity to ensure quality accurate, reliable, and safe diagnostic testing.

## Materials and methods

### A. EQA program operating guidelines and implementation policies

The COVID-19 EQA program design and enrollment were conducted from January-March 2021 and was informed by several variables and public health strategies including testing site location, testing site type and client base, testing volume per site, and data implications. The EQA program was designed to be ongoing as public health needs dictated, with a total of 6 rounds of challenges per year sent to sites every 2 months. CMPT is a not-for profit quality assurance service operated from the Department of Pathology and Laboratory Medicine at the University of British Columbia. Research and development costs for developing the program were paid through CMPT central funds and BCCDC central funds. Ongoing operating costs for continual program implementation were recovered through participant- paid fee for service subscription. Sites enrolled in CMPT’s EQA programs through CMPT’s website. Subscribers then were given access to the private and confidential data portal for the entering of results, viewing of result letters, and final reports.

Due to the high demand for this EQA program and the high volume of sites enrolled in a short period of time, CMPT adapted their data management software to enable near real-time data analysis, and results reporting. The system was also modified so that accreditation bodies would receive the results of testing sites in their catchment area within 24 hours. A working policy was adopted by all parties that should a site underperform either as a single test event or serially, that CMPT would provide site support including mentoring, creating online tutorials, and providing technical support as needed. The eight HAs in BC also offered training, re-testing, and monitoring of activities as a way to improve testing performance. DAP retained responsibility for following up with sites for failed EQA challenges and request probable cause explanation.

### B. EQA program implementation design

Importantly, although the EQA program was launched with POCT sites as the intended client base, the formulation of each survey/challenge set was designed and developed for site use by either NAT and antigen-based testing applications (see Methods, Section C below). Clinical respiratory sample collection methods were also considered (nasopharyngeal and oropharyngeal methods). Each of the 6 challenge sets consisted of four blinded (Content of each vial was unknown to the testing operator) samples packaged in leak-proof vials, with each vial including either SARS CoV-2 positive or negative samples as shown in Table 1. The rationale for sending positive samples of varying viral concentrations was informed by the reality that several commercial test methods were being utilized concurrently across BC and Canada, each with varying use protocols and intrinsic test performance characteristics (including sensitivity and specificity). With this design, the participating site’s testing performance (e.g. accuracy, reliability) could be objectively evaluated regardless of the testing method being used.

**Table 1:** An example of a SARS-CoV-2 EQA survey set.

Sample 1	Sample 2	Sample 3	Sample 4
Positive (medium)	Negative	Positive (strong)	Positive (strong)

Sample types were randomized and blinded by a unique survey number for each challenge round/shipment. Samples were labeled and identified by “COV”, followed by the year and month, e.g. COV2211-1, COV2211-2, COV2211-3, COV2211-4 (survey shipped November 2022), and also marked by different colors to prevent confusion between survey samples when testing. Participants were allotted two weeks to run the samples and submit their results (see Methods, Section C below).

### C. Sample preparation and validation

CMPT and BCCDC collaborated in the formulation of the PT challenges under an institutional material transfer agreement, with all base source material provided by the BC Centers for Disease Control. SARS-CoV-2 virus (Wuhan) was cultured in African green monkey kidney epithelial cells using a positive clinical sample obtained by BCCDC through routine surveillance. Plaque purification methods were used to culture virus to high titre at the BCCDC containment Level 3 (CL-3) laboratory in Vancouver, Canada. Titre and viral supernatant were evaluated for purity by qRT-PCR and stored at -80°C in CL-3 prior to further manipulation. The undiluted virus culture material had a concentration of 6.0 x 10^4^ TCID50/mL and a CT value of 13.

The stock virus was diluted with Dulbecco’s Modified Eagle’s Medium (DMEM) (Invitrogen, ThermoFisher Scientific) to 3.0 x 10^3^ TCID/mL and 1.2x10^3^ TCID50/mL, and denoted as strong positive and medium positive samples, respectively. The strong positive sample had a CT value of 15 and the medium positive sample had a CT value of 19. Following stock titration, live virus cultures were treated with β-propiolactone (BPL) for inactivation and safe transport to the CMPT laboratory. All further manipulation with non-infectious material was conducted under CL-2 conditions at CMPT. Because some nucleic acid detection methods use the Ribonuclease P (RNase P) housekeeping gene to validate the integrity of the RNA in the sample as an internal control, negative control samples were initially made by collecting saliva from healthy donors. However, a more consistent and sustainable negative sample was formulated later in the program by using adenocarcinomic human alveolar basal epithelial (A549) cell lysate (2 million/mL); this sample formulation had a CT value for RNase P of approximately 24. Negative samples were prepared prior to manipulating positive samples to avoid cross-contamination. All samples were prepared and stored at -70°C one week prior to the shipment date.

Aliquots of 100μL of the sample type (Table 1) were dispensed into 2mL O-ring vial (Axygen, Corning, AZ). On the shipment date onwards, samples were stored at room temperature to mimic the transit and storage conditions of the majority of testing laboratory conditions. Samples quality control (QC) was performed using the Abbot Panbio Rapid antigen kit. (Abbott Park, Illinois, USA). CMPT’s PT panels undergo rigorous stability testing to ensure challenge quality up to at least 21 days post shipment. Stability of the COVID-19 samples was monitored on the shipping date (Day 0), one week after shipping (Day 7), and on the due date (Day 14). Stability of the sample set was also tested again in-house on the day CMPT received the last report to ensure sample behaved as expected.

Participants were provided with detailed written instructions for sample processing and results reporting. Sites were instructed to swab the sample tube aliquot as they would a clinical sample and process it as a simulated nasopharyngeal swab collected from a patient. Each vial was processed individually, for a total of four simulated nasopharyngeal swabs. Participants were asked to evaluate the sample as per their site’s protocol. A help line was available to assist customers with troubleshooting and questions. Laboratories were instructed to contact CMPT if they had any questions about the sample processing or reporting of results. CMPT provided technical support and help as needed within the limits of the requirements of an EQA provider.

### D. Data entry and analysis

Results reporting was required no later than 14 days post shipment date, which accommodated the BC provincial policy and desire for near real-time quality oversight of testing. All results were entered using the CMPT’s members online portal [13] which is secured by unique user log- in firewalls and accessible only to CMPT EQA programs subscribers. Each site was asked to include the following information: date of sample receipt, date of testing, site/instrument identification, reporting person, testing method, and results. A comments section was also available for laboratories to add if needed. CMPT did not collect information about the testing site type (e.g. laboratory or community-based sites). Immediately after results submission, participants received individual report letters summarizing their results and grades received; a copy of this result letter was sent to the accreditation body (DAP or other agency) for review as per policy. Laboratories were informed beforehand their information would be shared with their accreditation agencies if applicable. After the 14-day survey deadline, the aggregate data was analyzed per testing method and summarized into a final report to allow for inter laboratory comparison of results; this report was uploaded to the member’s portal for all participants to see. Interlaboratory comparison is enabled however individual site identifiers are not listed to support anonymity. Testing sites that did not report results within the due date graded “unacceptable”.

### E. Statistical analysis

In order to account for the clustering (dependencies) within the testing sites because there are multiple samples of the same type in a survey, as well as within each site across their repeated surveys, Generalized Estimating Equations (GEE) for logistic regression was used to analyze a binary outcome of Acceptable/Unacceptable. The model was fit to the outcome with Test, Sample, and Survey as the independent/explanatory variables (fixed effects) and included the two-way interactions between test and each of the other two variables (sample and survey). The testing site was used in the model to identify the groups or clusters for the data that comes from the same testing site both within each survey and across surveys.

## Results

### Number of testing sites

**Fig 1:**
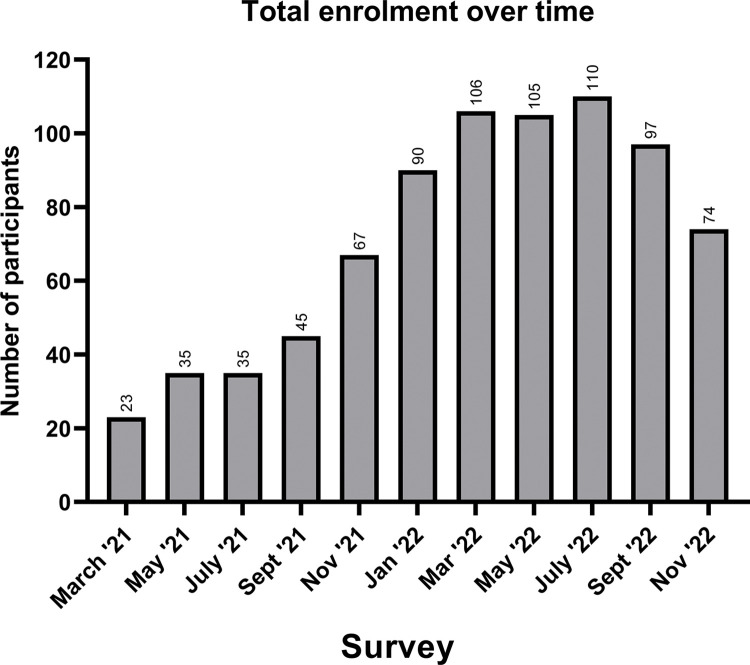
Total number of EQA participants from March 2021 to November 2022. This chart indicates the enrolled participants and the growth in the number of participants from the first survey in March 2021, to the most recent survey in November 2022. Peak enrolment was observed in July 2022.

Subscription in the new COVID-19 EQA program grew significantly over the course of the first year and included a valuable representative sample from the public and private sectors, types of testing facilities, geographic locations and population served (Fig 1). The number of participants increased by 3.2-fold within a year. In 2021, 6 high- volume laboratory hubs (located within 6 different HAs in BC) participated, as did 91 private sector POCT sites across Canada between the period of March 2021 to November 2022. On the public sector side, an average of 30 testing sites per survey received PT challenges, these were mainly long-term care facilities conducting daily routine testing and screening. Program enrollment reflected community population density and testing prioritization across the province of BC well with 57% of PT program participants located within the metropolitan Vancouver area and 27% of participants enrolled from Northern, Interior, and Vancouver Island regions; 16% of program subscriptions were from other provinces across Canada.

### Testing site performance

**Fig 2:**
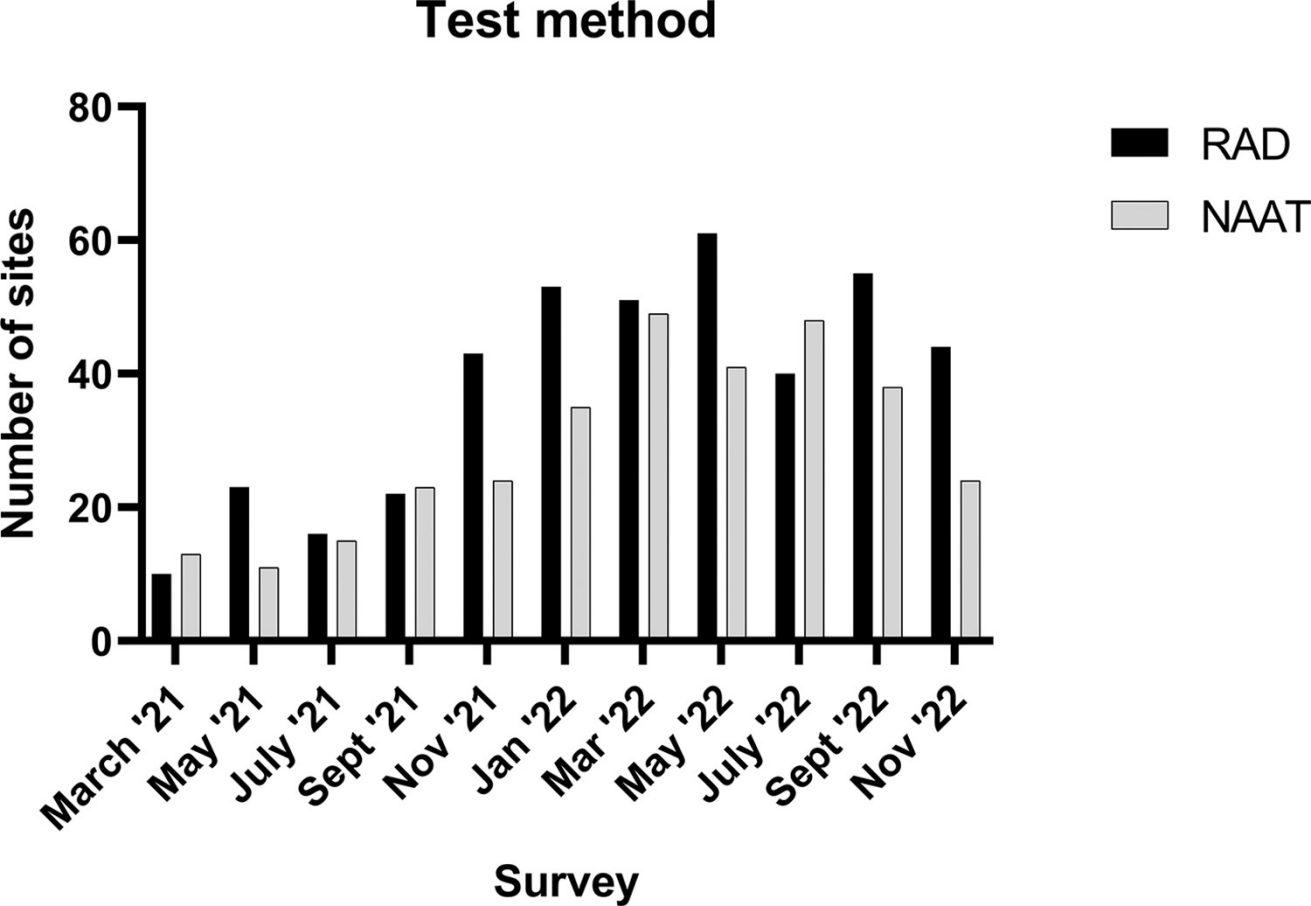
Distribution of sites utilization of each primary testing methods from March 2021 to November 2022. This chart shows the growth of participating sites per testing method, with peak enrollment of testing sites utilizing the RAD method in May 2022.

**Fig 3:**
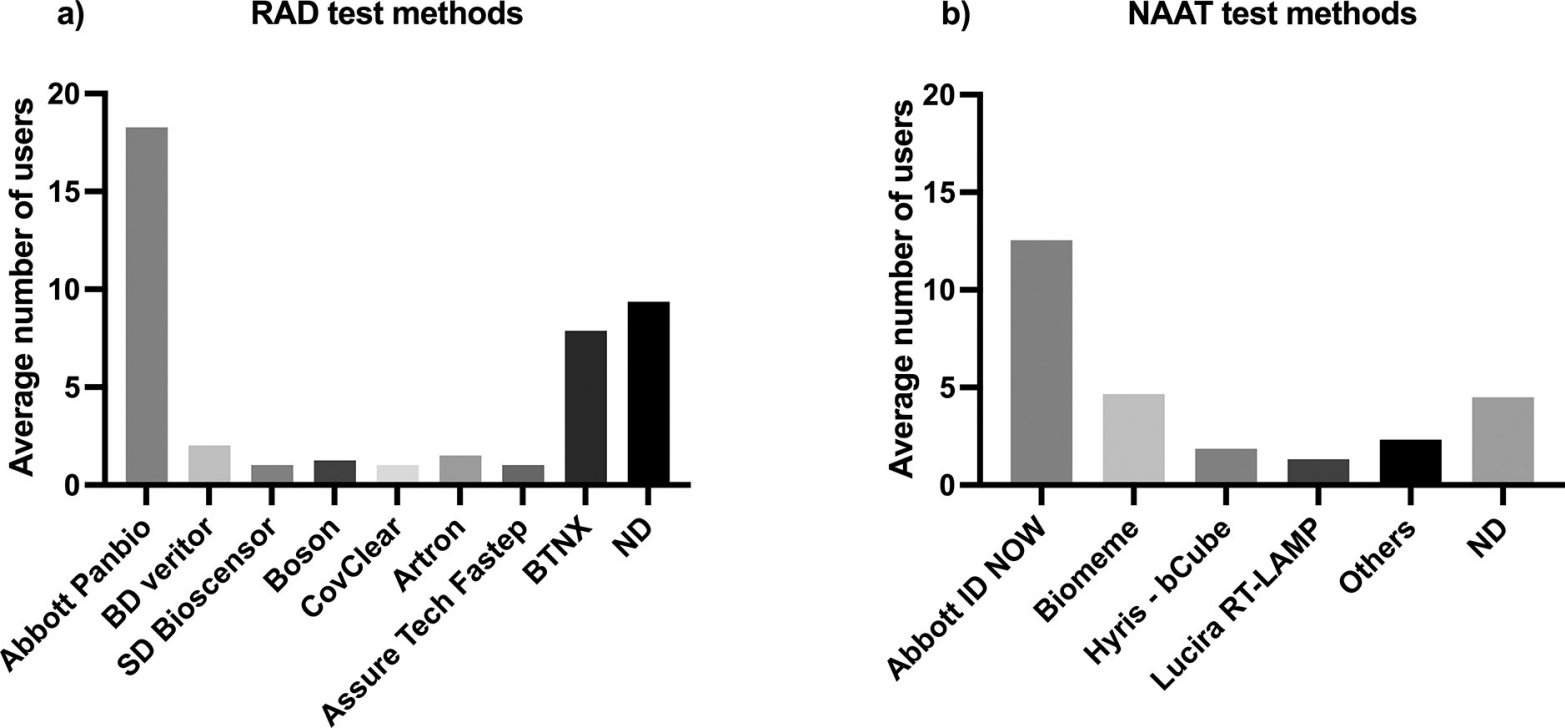
Distribution of test types for RAD and NAT used over the period of March 2021 to November 2022. **(a)** The total number of RAD test types, the most common test type was the Abbott Panbio kit. **(b)** The distribution of NAAT test types, the most common type was the Abbott ID NOW kit. The charts show the total number of uses of each test type and the frequency of each test used.

Testing methods employed by sites varied between participants using either RAD or NAT (Fig 2). The most frequently used commercial RAD test was the Abbott Panbio kit (Fig 3A) and the most frequently used commercial platform for NAT was the Abbott ID Now (Fig 3B).

The number of acceptable results obtained by testing sites using the RAD methods was low for surveys 1 and 2 (46% and 58%) but eventually increased to 91% result accuracy by January 2022. This performance trend persisted at >90% results accuracy throughout 2022 (Table 2). The percentage of acceptable results obtained by sites using NAT was consistently above 90% throughout the entire study period.

**Table 2:** 2021–2022 EQA results–Overall performance per survey.

Survey	Total testing sites	Rapid Antigen Test		Nucleic Acid Tests	
		No. of participants	Correct report (%)	No. of participants	Correct report (%)
March 2021	23	10	46	13	95
May 2021	34	23	58	11	97
July 2021	31	16	98	15	91
September 2021	45	22	83	23	91
November 2021	67	43	67	24	98
January 2022	88	53	91	35	96
March 2022	100	51	99	49	100
May 2022	102	61	98	41	99
July 2022	88	40	98	48	98
September 2022	93	55	98	38	100
November 2022	68	44	95	24	97

### Negative samples

Results reported using either RAD or NAT testing methods were consistent with the expected results for the negative sample type across the period analyzed and sites performed consistently well overall (Tables 3 and 4). Utilization of either RAD or NAT methods was equally as likely to produce a true negative test result.

**Table 3:** Comparing COVID-19 test methods employed for the estimated probability of achieving an acceptable test result for negative samples only.

Test	Survey	Estimated probability	*SE
RAD	1	0.923	0.045
NAT	1	0.979	0.021
RAD	2	0.946	0.029
NAT	2	0.976	0.029
RAD	3	1	0
NAT	3	0.934	0.04
RAD	4	0.975	0.013
NAT	4	0.924	0.049
RAD	^6	0.986	0.011
NAT	6	0.969	0.012
RAD	7	0.999	0.001
NAT	7	1	0
RAD	8	0.997	0.004
NAT	8	0.994	0.005
RAD	9	0.998	0.001
NAT	9	0.995	0.005
RAD	10	0.999	0.001
NAT	10	1	0
RAD	11	0.997	0.002
NAT	11	1	0

*SE = Standard error

^ Note that no negative samples were included in the 5^th^ survey

**Table 4:** Comparing COVID-19 test methods employed for the estimated probabilities of achieving an acceptable test result with a 95% confidence interval (asymp.LCL, asump.UCL), by individual survey for the negative samples only.

Survey	Estimated probability	SE	asymp.LCL	asymp.UCL	z.ratio	*p.value
1	-0.056	0.05	-0.195	0.084	-1.121	1
2	-0.03	0.041	-0.144	0.085	-0.721	1
3	0.066	0.04	-0.047	0.178	1.632	1
4	0.051	0.05	-0.091	0.193	1.011	1
^6	0.018	0.016	-0.028	0.063	1.102	1
7	-0.001	0.001	-0.004	0.002	-1.11	1
8	0.003	0.007	-0.016	0.021	0.384	1
9	0.004	0.005	-0.012	0.019	0.673	1
10	-0.001	0.001	-0.004	0.002	-1.202	1
11	-0.003	0.002	-0.01	0.003	-1.493	1

*Confidence level adjustment and *p* value adjustment using the Bonferroni method for 10 tests

^ Note that no negative samples were included in the 5^th^ survey

### Medium positive samples

**Fig 4:**
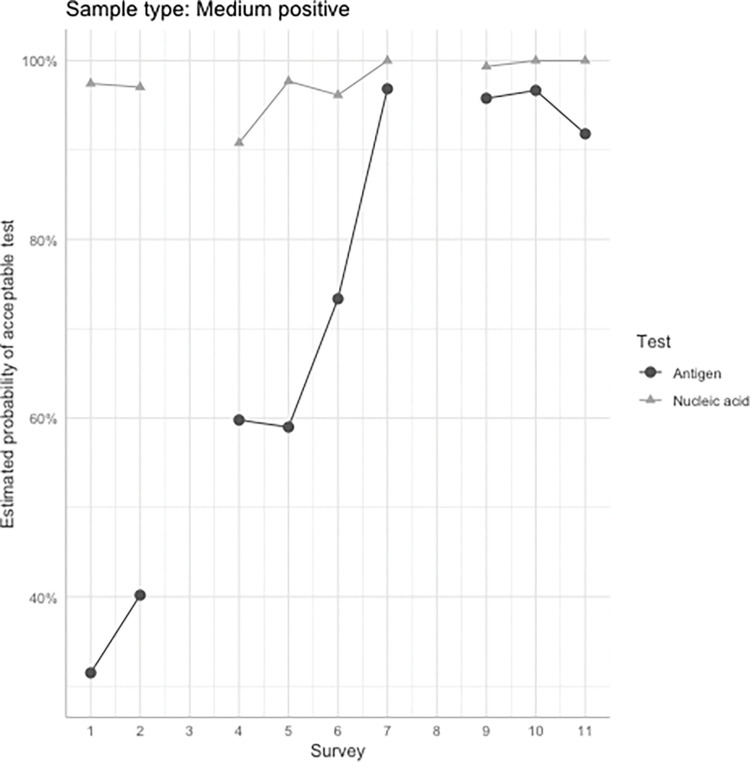
Overall trend of acceptable results reported across surveys for the medium positive sample. This graph depicts the upward trend in acceptable results over time for the RAD methods compared to the NAT methods.

Results reported using NAT methods were highly consistent with the expected results for medium positive samples however, sites using RAD testing methods showed poorer performance at the beginning of the program with many samples falsely reported as negative (Tables 5 and 6). Performance of sites using RAD testing methods improved over time (Fig 4). In summary, there was a statistically significant difference between the two testing methods regarding the probability of achieving acceptable results for the medium positive samples in surveys 1, 2, 5, and 6 (Table 6).

**Table 5:** Comparing COVID-19 test methods employed for the estimated probability of achieving an acceptable test result (estimate) for medium positive samples.

Test	Survey	Estimated probability	*SE
RAD	1	0.315	0.125
NAT	1	0.974	0.025
RAD	2	0.402	0.092
NAT	2	0.97	0.026
RAD	^4	0.598	0.102
NAT	4	0.908	0.06
RAD	5	0.59	0.075
NAT	5	0.977	0.021
RAD	6	0.733	0.071
NAT	6	0.962	0.025
RAD	7	0.968	0.023
NAT	7	1	0
RAD	^9	0.958	0.029
NAT	9	0.993	0.007
RAD	10	0.967	0.023
NAT	10	1	0
RAD	11	0.918	0.039
NAT	11	1	0

*SE = Standard error

^No medium positive samples were sent in surveys 3 and 8.

**Table 6:** Comparing COVID-19 test methods employed for the estimated probability of achieving an acceptable test result with a 95% confidence interval (asymp.LCL, asump.UCL), by individual survey for the medium positive samples only.

Survey	Estimated probability	SE	asymp.LCL	asymp.UCL	z.ratio	*p.value
1	-0.66	0.128	-1.015	-0.304	-5.147	<0.00001
2	-0.569	0.097	-0.836	-0.301	-5.885	<0.00001
^4	-0.31	0.118	-0.638	0.018	-2.619	0.07935
5	-0.387	0.078	-0.602	-0.172	-4.991	<0.00001
6	-0.228	0.076	-0.438	-0.018	-3.017	0.02296
7	-0.032	0.023	-0.095	0.032	-1.369	1
^9	-0.036	0.03	-0.119	0.048	-1.185	1
10	-0.033	0.023	-0.098	0.031	-1.433	1
11	-0.082	0.039	-0.191	0.027	-2.088	0.331

^ No medium positive samples were sent in surveys 3 and 8.

*Confidence level adjustment and *p* value adjustment using the Bonferroni method for 10 tests.

### Strong positive sample

Results reported using the RAD and NAT methods were consistent with the expected results for the strong positive sample type across the 11 surveys analyzed (Tables 7 and 8).

**Table 7:** Comparing COVID-19 test methods employed for the estimated probability of achieving an acceptable test result (estimate) for strong positive samples.

Test	Survey	Estimated probability	*SE
RAD	1	0.862	0.073
NAT	1	0.97	0.033
RAD	3	1	0
NAT	3	0.906	0.05
RAD	4	0.953	0.025
NAT	4	0.892	0.043
RAD	5	0.951	0.022
NAT	5	0.973	0.034
RAD	6	0.974	0.014
NAT	6	0.954	0.028
RAD	7	0.998	0.002
NAT	7	1	0
RAD	8	0.993	0.006
NAT	8	0.991	0.01
RAD	9	0.997	0.002
NAT	9	0.992	0.009
RAD	10	0.997	0.002
NAT	10	1	0
RAD	11	0.993	0.004
NAT	11	1	0

*SE = Standard error

**Table 8:** Comparing COVID-19 test methods employed for the estimated probability of achieving an acceptable test result with a 95% confidence interval (asymp.LCL, asump.UCL), by individual survey for the strong positive samples only.

Survey	Estimated probability	SE	asymp.LCL	asymp.UCL	z.ratio	*p.value
1	-0.108	0.08	-0.332	0.116	-1.35	1
3	0.094	0.05	-0.047	0.234	1.874	0.6096
4	0.061	0.049	-0.077	0.199	1.239	1
5	-0.021	0.04	-0.134	0.091	-0.532	1
6	0.02	0.031	-0.068	0.107	0.629	1
7	-0.002	0.002	-0.008	0.003	-1.235	1
8	0.002	0.012	-0.032	0.036	0.176	1
9	0.005	0.009	-0.021	0.031	0.495	1
10	-0.003	0.002	-0.008	0.003	-1.313	1
11	-0.007	0.004	-0.017	0.004	-1.693	0.9038

*Confidence level adjustment and *p* value adjustment using the Bonferroni method for 10 tests.

For each of the surveys there is no evidence that the probability of an acceptable test for the strong positive samples is different between the test methods.

## Discussion

Clinical diagnostic testing is critical for effective patient care and treatment and testing data significantly influences public health as we have observed across the world during the COVID-19 pandemic and the rapid scale up of diagnostic testing and screening [14]. All diagnostic testing requires consistent and objective external quality assurance oversight and evaluation to ensure test results are accurate, timely, reliable, and help prevent unnecessary harm to patients. EQA programs that incorporate PT [15] into the assessment process are essential elements of quality assurance programing and regulation. Our experience as an ISO 17043-accredited EQA provider, supporting the quality assurance during rapid scale up of COVID-19 testing and screening in Canada demonstrates this enormous value, especially when a myriad of testing methods and testing site types are involved. This study spanned nearly two years and included 11 individual surveys.

When comparing acceptable results reported for both RAD and NAT methods relative to the sample type using GEE for logistic regression over the course of 11 surveys, no statistical significance was found between NAT and RAD methods when analyzing the negative and strong positive samples. However, NAT methods had a significantly higher probability of producing acceptable results when compared to RAD methods (p<0.01) for medium positive samples for surveys 1, 2, 5, and 6 (Table 6). In the launch of this new program, inclusion of testing sites from both public and private sectors as well as testing location type, testing volume, and population service area added important variables that enabled valuable information for quality monitoring and near-real time intervention. It was evident that the performance of testing sites utilizing RAD testing methods significantly varied when challenged with a medium positive sample. However, the number of false negative results substantially diminished over time as shown in Table 6 and Fig 4, indicating the improvement of performance.

The high percentage of correct results (98%) in July 2021 (survey 3), was most likely a consequence that no medium positive samples were included in the survey, while the reduction in performance observed in November 2021 (survey 5) was most likely due to the inclusion of 3 medium positive samples in the survey (Table 2). The percentage of correct results using NAAT was consistently above 90% throughout this survey period. We also provide evidence here that the high frequency design of the PT surveys (6 times per year) allowed for early intervention with testing sites, and provided valuable information showing the progression of testing performance for the laboratories and community-based sites.

Although the typical frequency of CMPT PT surveys is usually two to three per year, the higher frequency of PT surveys in this study (every two months) better enabled the rapid identification of problems in the testing for COVID-19 and interpretation of results soon after POCT sites starting testing (and to high volumes) and releasing results. This allowed our collaboration group (reference laboratories, health authorities, and accreditation bodies) to implement quick interventions at different levels and by different quality partners to correct and improve the quality of results reported by POCT sites.

Our experience during the period from March 2021 to November 2022 revealed important limitations and issues to consider in situations where the diagnostic testing and thus, PT, is taken outside the typical clinical laboratory setting. Upon investigation and in consultation with testing sites, it was noted that some sites would report weakly positive bands as “negative”. In some cases, sites would have problems following instructions on how to process the samples. These sites were identified and given additional support to rectify the incorrect results and procedures. We believe that these interventions influenced the sites towards improving their performance through surveys 1 through 5 and trend of >90% results accuracy continued for the remainder of the testing period (surveys 6–11) (Fig 4).

One issue is the lack of awareness of the potential value of PT for educational purposes and the value of participating in a program for testers. In a survey conducted by CMPT in February 2021 [16], one third of the responders indicated they had never heard about PT and were even less aware of the process and requirements of EQA. In order to address this challenge, CMPT offered continual support to clients, coaching them on PT testing, interpreting their results, and supporting them to successfully achieve and maintain accreditation. Another challenge that appeared early on originated from the PT provider side of the program. Instructions for the processing of samples are usually geared to a trained and experienced laboratory technologist and early results from non-laboratory sites suggested a problem with lack of clarity in CMPT’s instructions. This showed to be a challenge after a couple of surveys, when instructions needed to be modified to be better understood by testing staff not familiar with conventional laboratory language.

This study revealed an important third consideration when planning a PT program for any kind of diagnostic testing. Our results from this EQA period indicated great performance for both RAD and NAT detection technologies when testing CMPT’s strong positive samples. Also, sites frequently underperformed when challenged with the medium positive sample, and so, the inclusion of a sample that was weaker than the normal QC in the testing kit (for RAD in particular) proved to be a very valuable tool to evaluate interpretation of results by the tester. Significant differences in performance were observed when comparing the results obtained by both RAD and NAT methodologies when testing the medium positive sample. Testing sites consistently struggled to get accurate results for this sample type when testing using RAD methods. In July 2021 when no medium positive samples were included, 98% of reports were correct however, in November 2021 when 3 out of 4 samples were samples only 67% of results were correctly reported. Coaching, training, and mentoring showed to improve the performance of these labs to reach the same level of performance than the labs testing by NAT methods by the end of the first year of the PT program.

Lastly, the rapid proliferation of testing technologies, presented a novel challenge to CMPT as a PT provider to be constantly adapting and to formulate samples that would mimic in appearance and behavior, the typical respiratory sample(s) and sampling methods used for COVID-19 testing. CMPT demonstrated versatility and creativity in the design of PT samples in order to serve the needs of the wide range of clients and testing methodologies.

Decentralized, lower complexity testing which is conducted at the community level, performed outside of the clinical laboratory, has become an increasing trend across the world and in certain cases has enabled and improved patient care and public health in certain critical global health emergencies such as HIV, TB and malaria. Rapid detection of newly infected HIV patients and immediate pharmaceutical intervention at the community level (“test and start”), in the singular disease example of HIV alone, has without question saved millions of lives, and remains a shining example of the impact the availability of low complexity testing diagnostic testing can have when made available at the community-level. However, robust post-marketing surveillance of test kit performance and testing site supervision including a robust EQA program are critical pillar of a government’s quality assurance program, especially when consequences of pharmaceutical treatment and impact to patient mental health can be severe [17].

In nearly every nation widely implementing COVID-19 testing, test performance of NAT tests for SARS-CoV-2 have consistently produced more accurate and reliable results than RADs and multiple EQA programs have provided valuable supporting data for this [18–20]. Several EQA programs for SARS-CoV-2 antigen testing have been developed alongside their respective national scale up responses and have been pivotal to provide information as a quality tool with which to monitor community-based testing [21–23]. Our work to develop and implement a new EQA program for SARS-CoV-2 here in Canada tailored to both NAT and RAD methods, adds to this body of experience from other EQA providers [24, 25], and reinforces the value of stakeholder collaboration for quality monitoring of diagnostic testing and in support of public health initiatives. Our EQA program and PT challenge design included a range of viral concentrations for each challenge (strong, medium, negative), enabling us to measure site accuracy with weakly positive results, a recurring issue with RAD methods.

Working closely with accrediting agencies and health authorities, testing sites performing both NAT and RAD received supportive mentoring and retraining when required. As our collaborative EQA program data demonstrates, testing performance can vary significantly between the types testing sites (e.g. laboratory hub versus long term care facility) and the types of methods and technologies they employ.

This EQA program enabled early monitoring of the test performance characteristics of many novel IVDs that newly entered the market, and enables the early detection of false negatives and false positives. This empowered quality partners with near real-time information with which to support testing sites with re-training, mentorship and coaching. Our experience with the weak performance for multiple months suggested that the complexity of RAD testing was under-rated, and that a more intense pre-performance training program could have been considered. In its absence it is unknown how many falsely negative test results were reported. EQA program data importantly, supported the post-marketing surveillance of several IVDs newly employed across Canada. Based on our experience with sites implementing RAD methods with various test performance characteristic, we strongly suggest EQA programs implement a similar range of challenge complexity.

## Conclusions

Diagnostic testing, regardless of where it is performed, requires external quality assessment and oversight to ensure testing accuracy and data quality. EQA providers have a unique and critical role to play in diagnostic testing quality oversight and assurance. By working in collaboration and partnership with health authorities and key stakeholders, and EQA partner can help support patient care and public health in emergency and non-emergency times. EQA programs should be intentionally designed to mimic clinical samples and account for a myriad of testing methods that may be employed at testing sites. PT challenges should employ a range of modalities (weak and strong signals) for rapid diagnostic testing methods such as lateral flow tests, where results are often reported as binary results: “positive or negative”. Thorough tester training and ongoing supportive supervision should be ensured when any diagnostic testing is performed outside of an accredited medical laboratory setting.

## Supporting information

S1 Data Testing results obtained per testing site, sample, and survey. (XLSX)
